# PrimPol Bypasses UV Photoproducts during Eukaryotic Chromosomal DNA Replication

**DOI:** 10.1016/j.molcel.2013.10.035

**Published:** 2013-11-21

**Authors:** Julie Bianchi, Sean G. Rudd, Stanislaw K. Jozwiakowski, Laura J. Bailey, Violetta Soura, Elaine Taylor, Irena Stevanovic, Andrew J. Green, Travis H. Stracker, Howard D. Lindsay, Aidan J. Doherty

**Affiliations:** 1 Genome Damage and Stability Centre, School of Life Sciences, University of Sussex, Brighton BN1 9RQ, UK; 2 Lancaster Medical School, Faculty of Health and Medicine, Lancaster University, Lancaster LA1 4YQ, UK; 3 Institute for Research in Biomedicine (IRB Barcelona), Barcelona 08028, Spain

## Abstract

DNA damage can stall the DNA replication machinery, leading to genomic instability. Thus, numerous mechanisms exist to complete genome duplication in the absence of a pristine DNA template, but identification of the enzymes involved remains incomplete. Here, we establish that Primase-Polymerase (PrimPol; CCDC111), an archaeal-eukaryotic primase (AEP) in eukaryotic cells, is involved in chromosomal DNA replication. PrimPol is required for replication fork progression on ultraviolet (UV) light-damaged DNA templates, possibly mediated by its ability to catalyze translesion synthesis (TLS) of these lesions. This PrimPol UV lesion bypass pathway is not epistatic with the Pol η-dependent pathway and, as a consequence, protects xeroderma pigmentosum variant (XP-V) patient cells from UV-induced cytotoxicity. In addition, we establish that PrimPol is also required for efficient replication fork progression during an unperturbed S phase. These and other findings indicate that PrimPol is an important player in replication fork progression in eukaryotic cells.

## Highlights

• PrimPol is a DNA primase-polymerase catalyzing bypass of UV and oxidative lesions
• PrimPol operates in a UV lesion tolerance pathway that is non-epistatic with Pol η
• PrimPol null cells are defective in fork progression, particularly after UV treatment
• Loss of PrimPol leads to increased mitotic chromosomal breaks

## Graphical Abstract

**Figure:**
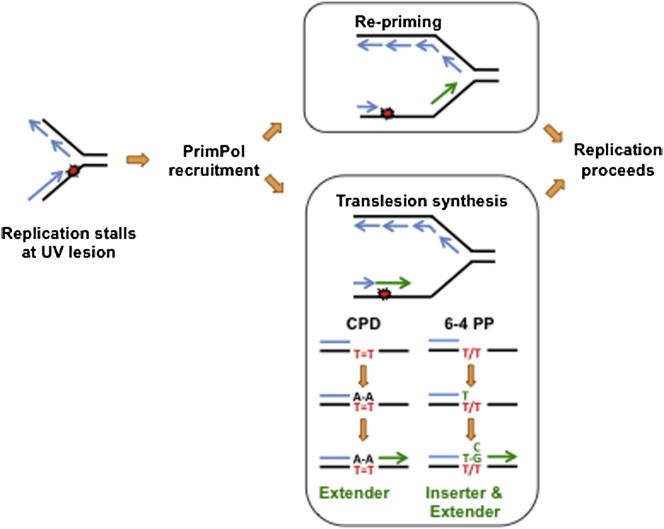

## Introduction

DNA damage frequently stalls the DNA replication machinery, and therefore bypass mechanisms exist to ensure complete chromosomal duplication and prevent genome instability (Aguilera and Gómez-González, 2008). DNA replication can restart directly at stalled replication forks using low-fidelity DNA polymerases that catalyze translesion synthesis (TLS; Sale et al., 2012) or by homologous recombination (HR), which utilizes an alternative undamaged sister template to allow extension of the stalled primer terminus (Li and Heyer, 2008). DNA replication can also proceed in the presence of damage by convergence of adjacent replicons, discontinuous synthesis of Okazaki fragments on the lagging strand, or repriming of DNA synthesis downstream of lesions on the leading strand (Heller and Marians, 2006, Yeeles and Marians, 2011). These produce single-stranded (ss) DNA gaps behind replication forks that are subsequently filled using TLS or HR (Lopes et al., 2006). Although our understanding of damage tolerance processes is advancing, identification of enzymes involved in these pathways is not yet complete. Eukaryotic cells employ a number of distinct DNA polymerases for propagation of chromosomal DNA, but only one archaeo-eukaryotic primase (AEP), Prim1, has been described as requisite (Frick and Richardson, 2001). While AEPs were once considered to solely prime DNA synthesis, it is now evident that AEPs have additional biological functions, exemplified by their role in prokaryotic DNA break repair (Della et al., 2004, Pitcher et al., 2007). Here, we establish that a second AEP is present in human cells, PrimPol (CCDC111), which can initiate DNA and RNA synthesis de novo and elongate existing DNA chains. Furthermore, we investigate cellular roles of PrimPol and establish that this AEP is required for efficient fork progression during chromosomal DNA replication on both damaged and undamaged templates in higher eukaryotes.

## Results

**Figure 1:**
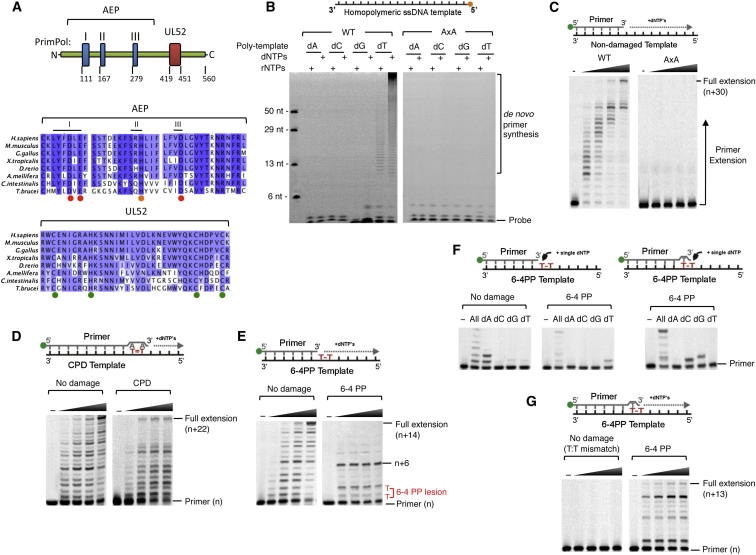
Domain Architecture and Catalytic Activities of Human PrimPol (A) Schematic and multiple sequence alignment of PrimPol conserved domains. The catalytic AEP domain containing three signature motifs (I, II, and III) and the UL52-like zinc finger domain are indicated, including amino acid number. Multiple sequence alignment was generated with a selection of PrimPol homologs; blue shading indicates ≥ 40% sequence identity, red circles indicate residues required for metal ion binding, orange circle for nucleotide binding, and green circles for chelation of zinc. (B) Primer synthesis by wild-type (WT) His-tagged human PrimPol and catalytic mutant (AxA). Homopolymer DNA templates (500 nM) were incubated with dNTPs or rNTPs (500 μM), magnesium ions, and WT or AxA PrimPol (1 μM) for 2 hr at 37°C. (C) DNA synthesis by PrimPol. Primer-template substrate (20 nM) and dNTPs (200 μM) were incubated with or without (−) PrimPol (WT or AxA; 50 nM) at 37°C for increasing times (2, 5, 10, 15 min). (D–G) DNA synthesis by PrimPol on templates containing either a T-T *cis-syn* cyclobutane pyrimidine dimer (CPD) (D) or a T-T pyrimidine (6-4) pyrimidone photoproduct (6-4 PP) (E–G) was compared to PrimPol DNA synthesis on undamaged templates using primer extension assays as described in (C). CPD is annealed opposite two 3′ terminal dA residues, thereby testing PrimPol extension opposite the lesion (D). 6-4 PP is at bases +1 and +2 of template relative to 3′ terminus of primer to test read-through (E) and in the presence of single dNTPs for a single 30 min reaction to test nucleotide incorporation opposite 3′ T (F, middle panel). Primer with 3′ terminal dT opposite 3′ T of 6-4 PP was used to test nucleotide incorporation opposite 5′ T of lesion (F, right panel) and, when all dNTPs included, extension (G). Note: undamaged template in (G) contains a 3′ terminal T:T mismatch.

To determine if additional AEPs reside in the eukarya, a bioinformatics search was undertaken of the available eukaryotic genomes for genes with homology to AEP-like primases. In addition to the replicative primase, Prim1, we detected a second AEP-like gene/protein in eukaryotes, called CCDC111, previously reported to be a putative AEP superfamily member (Iyer et al., 2005). Following primase nomenclature, we named this enzyme PrimPol (Primase-Polymerase) to reflect its intrinsic biochemical activities (see below). PrimPol is present across the majority of eukaryotic species, from the earliest unicellular organisms (protists and algae) to plants, animals, and mammals, while some lower eukaryotes encode multiple PrimPol homologs. However, it is absent from prokaryotes, archaea, *Drosophila*, all but one fungal species, and *C. elegans*. PrimPol contains two discernable motifs (Figure 1A), an N-terminal AEP catalytic domain and a C-terminal zinc finger similar to the viral UL52 primase domain.

To determine the activities encoded by human *CCDC111*, we overexpressed recombinant PrimPol in *E. coli* and purified it to near homogeneity (Figure S1A). To test if it possessed DNA/RNA primase activity, we incubated PrimPol with homopolymeric ssDNA, nucleotides (NTPs or dNTPs), and magnesium. PrimPol synthesized primers in the presence of either NTPs or dNTPs on a poly(dT) template (Figure 1B), consistent with the requirement of eukaryotic AEPs for templated pyrimidines to catalyze dinucleotide synthesis (Frick and Richardson, 2001). Notably, dNTPs were utilized with a much greater efficiency than NTPs (Figure 1B). Mutation of the conserved active site metal binding residues (motif I: D114A, E116A; AxA) ablated priming activities (Figure 1B). Thus, PrimPol has robust DNA-dependent DNA and less prominent RNA primase activity, which is unique for eukaryotic primases. Although the eukaryotic replicative AEPs are dedicated DNA primases, some bacterial and archaeal AEPs are also DNA polymerases (Bocquier et al., 2001, Lipps et al., 2003, Della et al., 2004). To test if PrimPol has any associated DNA polymerase activities, the enzyme was incubated with a template-primer DNA substrate, dNTPs and magnesium. PrimPol readily extends the primer strand in a template-dependent manner in the presence of dNTPs (Figure 1C). The active site mutant also lacked polymerase activity (Figure 1C), confirming that both priming and extension activities are catalyzed by the same active site. To reflect the capacity of this enzyme to catalyze both DNA-dependent RNA/DNA primase and DNA polymerase activities, it was renamed PrimPol.

As bacterial AEPs are involved in DNA repair processes (Della et al., 2004, Pitcher et al., 2007), we assayed PrimPol for DNA lesion bypass activity. PrimPol efficiently bypassed 8-oxo-guanine lesions (Figure S1B), incorporating dA or dC with equal efficiency (Figure S1C), but was incapable of incorporating nucleotides opposite templated abasic or thymidine glycol lesions (Figure S1B). PrimPol was incapable of reading through a templated *cis-syn* thymine-thymine (T-T) cyclobutane pyrimidine dimer (CPD) (Figure S1B), an UV photolesion that is a potent block for cellular replicases (Figure S1D). However, PrimPol was capable of extending a primer terminus with two dA residues annealed opposite the T-T CPD, with an efficiency comparable to an undamaged TT (Figure 1D). In contrast, human Pol ε or an archaeal PolB replicase were incapable of extending this primer terminus annealed to a CPD (Figure S1E). UV light also generates T-T pyrimidine-pyrimidone photoproducts (6-4 PPs) in DNA, which are highly distorting and cannot be efficiently bypassed by any individual mammalian DNA polymerase in vitro. Although both Pol ε and PolB stalled at this lesion (Figure S1F), strikingly, PrimPol could incorporate opposite and extend from a templated 6-4 PP (Figure 1E). Single incorporation assays revealed that PrimPol-dependent bypass was error prone, incorporating dT opposite the 3′ T, and dG or dC opposite the 5′ T of the photoproduct (Figure 1F). Incorporation of dT opposite the 3′ T of a 6-4 PP may be a distinct mechanism employed by PrimPol to facilitate bypass, as primer extension was only observed in the presence of the 6-4 PP (Figure 1G). Together, these data establish that PrimPol is a competent TLS polymerase, bypassing specific oxidative and UV-induced DNA lesions.

**Figure 2:**
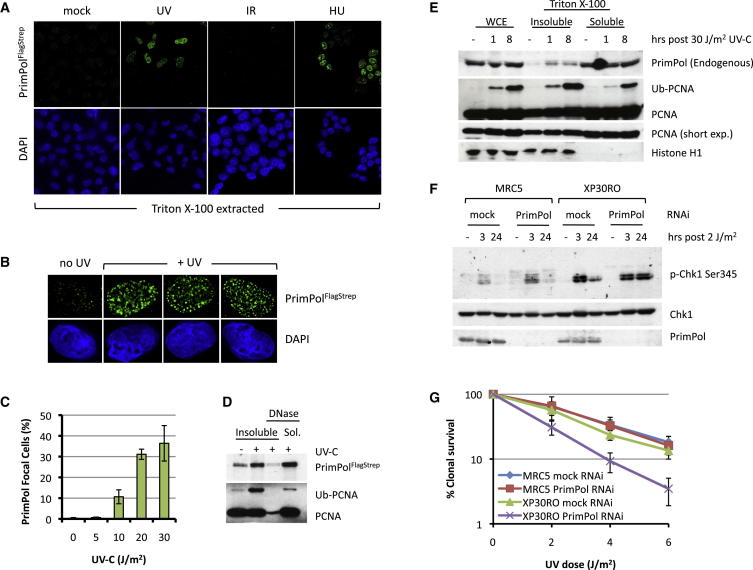
PrimPol Is Required for Tolerance of UV Photoproducts in a Pathway Independent of Pol η (A) Human (HEK293) cells stably expressing PrimPol with a C-terminal Flag-Strep-II tag (PrimPol^FlagStrep^) were either mock, UV-C (30 J/m^2^), or X-ray (5 Gy) irradiated or treated for 6 hr with hydroxyurea (HU; 10 nM); following recovery (1 hr for UV-C, 30 min for X-ray, immediately after HU treatment), cells were detergent extracted (0.5% Triton X-100) prior to immunofluorescent (IF) analysis with an anti-PrimPol antibody and DAPI counterstaining. (B) Representative images of nuclei containing detergent-resistant PrimPol foci. (C) Proportion of cells in which PrimPol assembled into foci was determined at varying UV-C doses following an 8 hr recovery; error bars indicate SD of three experiments, > 200 cells counted for each dose. (D) Mock or UV-C irradiated (30 J/m^2^) cells were allowed to recover for 8 hr before the Triton X-100 (0.5%) insoluble material was collected by centrifugation and treated with DNase and further centrifugated; the resulting samples were analyzed by western blot with anti-PrimPol and PCNA antibodies. (E) Normal human (MRC5) fibroblasts were either mock (−) or UV-C (30 J/m^2^) irradiated and, following recovery, were separated into Triton X-100 (0.5%) soluble and insoluble material and analyzed by western blot along with whole-cell extract (WCE). (F) Normal (MRC5) fibroblasts or XP-V (XP30RO) patient cells were either mock or PrimPol siRNA treated and mock (−) or UV-C (2 J/m^2^) irradiated and allowed to recover before cell lysates were prepared and analyzed by western blot to determine levels of phosphorylated Chk1 on Ser345. (G) UV-C clonogenic survival assays were performed with MRC5 and XP30RO cells either mock or PrimPol siRNA treated. Error bars denote SD of three experiments.

To establish the cellular localization of PrimPol, immunofluorescence (IF) detection of stably expressed PrimPol in HEK293 cells (Figures S2A–S2C) or endogenous PrimPol in 143B cells (Figure S2D) showed that it was predominantly cytoplasmic, although a small portion of this protein appeared nuclear. As PrimPol can bypass UV-induced DNA lesions in vitro, we next examined the subcellular localization of PrimPol in UV-irradiated cells. Cells stably expressing PrimPol were exposed to UV-C radiation and detergent-extracted prior to IF analysis. PrimPol relocalized to detergent-resistant subnuclear foci following UV-C irradiation (Figure 2A), numbering between 50 to several hundred per cell (Figure 2B), and the proportion of cells containing PrimPol foci was UV-C dose dependent (Figure 2C). Foci were also observed following nucleotide deprivation by hydroxyurea (HU) treatment (Figure 2A); however, this relocalization of PrimPol was not a general response to DNA damage, as no focal accumulation was observed following treatment of cells with ionizing radiation (IR) (Figure 2A), which induces DNA strand breaks. To confirm that UV-C-induced PrimPol foci represented sites of chromatin association, the detergent-insoluble chromatin fraction was prepared from UV-C-irradiated cells and treated with DNase. This treatment successfully solubilized monoubiquitinated PCNA, present at replication forks stalled at UV photoproducts (Kannouche et al., 2004), and also released PrimPol into the soluble fraction (Figure 2D). Endogenous PrimPol in normal human fibroblasts also relocalizes following UV-C irradiation, partitioning to the detergent-insoluble chromatin-enriched fraction (Figure 2E). Together, these data support a role for PrimPol in the cellular response to UV-C radiation.

Defects in the bypass of UV photoproducts during chromosomal DNA replication result in activation of the intra-S phase checkpoint due to replication fork stalling. This is particularly evident in xeroderma pigmentosum variant (XP-V) cells (Bomgarden et al., 2006, Despras et al., 2010), which lack the CPD TLS polymerase Pol η (Johnson et al., 1999, Masutani et al., 1999). As PrimPol bypasses 6-4 PPs and extends from CPDs, in addition to being a primase with a possible role in repriming downstream of lesions, we hypothesized that it may function in a damage tolerance pathway that is complementary to Pol η. Therefore, the effects of depleting PrimPol in normal and XP-V cells treated with UV-C were investigated. RNAi-treated cells were UV-C irradiated and, following recovery, analyzed for detergent-resistant RPA foci and activation of the intra-S phase checkpoint kinase Chk1. These RPA foci are indicative of RPA-coated ssDNA present at stalled replication forks and, consistent with a requirement for PrimPol to bypass UV photoproducts, a significant increase in focal cells was observed in PrimPol-depleted fibroblasts (Figure S2E). Concurrently, an increase in phosphorylated Chk1 (S345) was also observed (Figure 2F), indicating activation by the replication stress response kinase, ATR. As bypass of UV photoproducts is already substantially impaired in XP-V cells, no further increase in RPA focal cells was observed in PrimPol-depleted XP-V cells (Figure S2E). However, Chk1 activation remained elevated 24 hr after irradiation with a low UV-C dose (Figure 2F), indicating a persistent defect in UV photoproduct bypass in the absence of both PrimPol and Pol η. In accordance with this, we observed ∼5-fold increase of chromatin-bound Rad51 (Figure S2F), indicative of increased HR-mediated rescue of stalled replication forks in the absence of both PrimPol and Pol η. Despite PrimPol’s role in the tolerance of UV photoproducts, PrimPol-depleted normal fibroblasts did not present any major UV-C sensitivity, and similarly, XP-V cells were only mildly sensitive to UV-C irradiation (Figure 2G). However, PrimPol depletion in XP-V cells rendered them synergistically sensitive to UV-C irradiation, decreasing the surviving fraction by up to a factor of 4 (Figure 2G). XP-V cells show greater sensitivity to UV when treated with low doses of caffeine (Arlett et al., 1975). To determine if this was due to inhibition of PrimPol, we repeated UV colony survivals on XP-V cells in the presence of 0.38 mM caffeine. Both XP-V cells and those depleted for PrimPol were more sensitive to UV than in the absence of caffeine (Figure S2G), suggesting that PrimPol is not the source of caffeine sensitivity in XP-V cells. To establish if PrimPol plays any roles in nucleotide excision repair (NER), we depleted PrimPol in NER-deficient XP-A cells and observed an increase in UV sensitivity (Figure S2H), indicating that PrimPol’s primary role is not in NER. Together, these findings establish that PrimPol operates in a UV lesion tolerance pathway that is nonepistatic with Pol η and that this pathway protects XP-V cells from UV-C cytotoxicity by compensating for the loss of Pol η.

**Figure 3:**
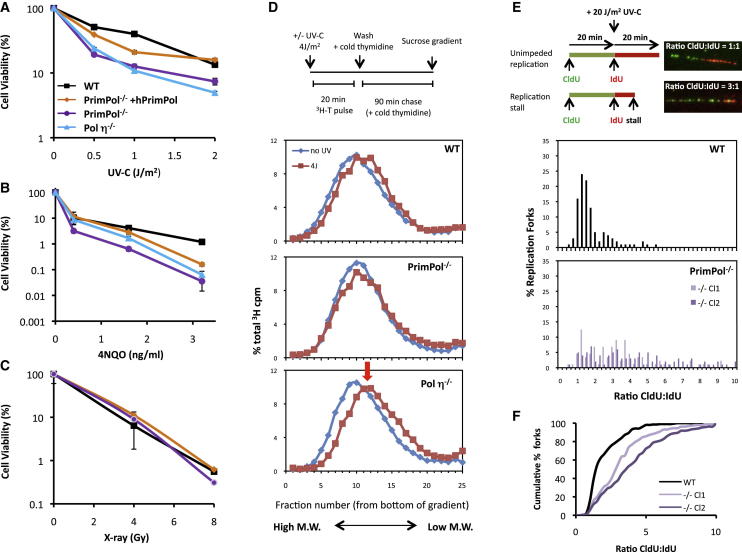
PrimPol Is Required for Replication Fork Progression on UV-Damaged DNA Templates in Vertebrate Cells (A–C) Viability of wild-type (WT) and DT40 knockout cell lines including PrimPol-deficient cells expressing human PrimPol protein (*PrimPol*^*−/−*^ + hPrimPol) was determined following exposure to UV-C (A), 4-nitroquinoline 1-oxide (4NQO; 48 hr treatment; B), and X-rays (C). Cells recovered for 48 hr after treatment before measurement of metabolic capacity. Error bars denote SD of three experiments, with two *PrimPol*^*−/−*^ cell lines used. (D) Alkaline sucrose sedimentation analysis of DNA from cells that were either mock or UV-C irradiated (4 J/m^2^) and immediately pulse-chased with ^3^H-thymidine. Representative of at least three experiments shown; red arrow indicates postreplication repair defect. (E) DNA fiber analysis of cells UV-C irradiated (20 J/m^2^) between the CldU and IdU labeling periods. CldU:IdU ratio distribution representative of two sets of experiments using two *PrimPol*^*−/−*^ cell lines (Cl1 and Cl2) is shown; > 100 DNA fibers scored for each. The average of these data is presented as a cumulative percentage of forks at each ratio (F). See Figure S3 for details on the knockout cells.

Next, we used avian DT40 cells to further investigate the role of PrimPol in the tolerance of UV-C-induced DNA damage. PrimPol knockout (*PrimPol*^*−/−*^) DT40 cells were viable (Figures S3A–S3C) and exhibited only minor proliferative (Figure S3D) and cell-cycle defects, with a delayed G2/M transit (Figure S3E). Consistent with PrimPol’s proposed role in the tolerance of UV photoproducts, *PrimPol*^*−/−*^ cells were sensitive to low doses of UV-C radiation and the UV mimetic 4-nitroquinoline 1-oxide (4NQO), in a similar manner to *Pol η*^−/−^ cells (Figures 3A and 3B), but were not sensitive to IR (Figure 3C). Expression of human PrimPol in *PrimPol*^*−/−*^ cells (Figure S3F) partially rescued both UV-C and 4NQO sensitivities (Figures 3A and 3B).

TLS-mediated damage tolerance can occur either at replication forks or in a postreplication manner. To determine the timing of PrimPol-mediated damage tolerance, the ability of *PrimPol*^*−/−*^ cells to perform TLS postreplicatively on daughter strand gaps was assessed using alkaline sucrose sedimentation analysis of nascent DNA in UV-C-irradiated cells (Lehmann, 1972). As expected, a postreplication repair defect was observed in *Pol η*^*−/−*^ cells, but no defect was observed in *PrimPol*^*−/−*^ cells (Figure 3D). This suggests that PrimPol is not required for postreplicative bypass of UV photoproducts. We next asked whether PrimPol is required to bypass UV photoproducts directly at stalled replication forks by analyzing DNA fiber spreads. Notably, in the absence of UV-C irradiation, overall replication fork speeds were reduced in *PrimPol*^*−/−*^ cells by ∼20% (Figure S3G), and a similar reduction in DNA replication was observed by monitoring incorporation of radiolabelled thymidine (Figure S3H), suggesting a role for PrimPol during unperturbed chromosomal DNA replication. To analyze fork progression on UV-damaged DNA, *PrimPol*^*−/−*^ cells were exposed to UV-C radiation between thymidine analog CldU and IdU labeling periods, and the ratios between the two labels were calculated. In mock-irradiated cells, this ratio was ∼1 (data not shown); however, following UV-C irradiation, this ratio increased significantly due to shortening of the second track, indicating an impediment in replication fork progression. Compared to wild-type cells, *PrimPol*^*−/−*^ cells showed a substantial increase in the spread and mean of CldU:IdU ratios (Figures 3E and 3F), indicating a marked increase in delay or blockage of replication fork progression on DNA containing UV photoproducts. In line with this, a reduction of radiolabelled thymidine incorporation was observed in *PrimPol*^*−/−*^ cells compared to wild-type cells at varying UV-C doses (Figure S3I). Together, these data demonstrate that PrimPol is required for efficient replication of UV-damaged chromosomal DNA in vivo, which is consistent with the ability of PrimPol to extend from CPDs and bypass 6-4 PPs in vitro.

**Figure 4:**
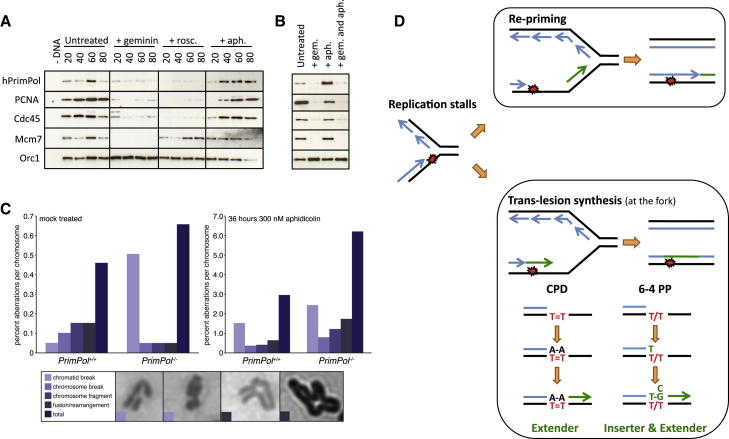
PrimPol Functions during an Unperturbed S Phase (A) His-tagged PrimPol (PrimPol at 12 ng/μl) was added to *Xenopus* egg extract supplemented with sperm nuclei. Extracts were treated with geminin (80 nM), roscovitine (0.5 mM), or aphidicolin (100 μg/ml) and incubated at 21°C. At the indicated times (minutes), chromatin was isolated and associated proteins subjected to western blot analysis with the antibodies indicated. (B) Experiment in (A) was repeated at a 60 min time point, and the last lane corresponds to a sample preincubated with geminin followed by aphidicolin treatment. (C) Analysis of metaphase aberrations in mock and aphidicolin-treated primary MEFs lacking PrimPol. Percentage and type of aberrations per chromosome are indicated. Examples of two chromatid breaks and two rearrangements from PrimPol-deficient cells are shown. See Figure S4 for details on the knockout cells. (D) Model of PrimPol-mediated replication fork progression. Following DNA replication stalling (depicted on the leading strand), PrimPol could reprime DNA synthesis downstream of the lesion to allow DNA replication to continue. PrimPol can also catalyze TLS of some DNA lesions and could directly extend the stalled primer terminus facilitating replication fork progression. With regards to UV-damaged templates, PrimPol could function in the error-free extension from CPDs and the error-prone bypass of 6-4 PPs.

The proliferative, cell cycle, and DNA replication defects observed in *PrimPol*^*−/−*^ cells (Figure S3) strongly support an additional role for PrimPol in normal S phase. To explore this further, we examined PrimPol’s localization on chromatin during a synchronous round of DNA replication in *Xenopus* cell-free egg extracts. Recombinant human PrimPol became bound to chromatin during an unperturbed S phase, and this binding was abolished when replication was inhibited prior to initiation by geminin or the CDK inhibitor roscovitine (Figure 4A). By blocking elongation with aphidicolin treatment, increased chromatin-bound PrimPol was observed (Figure 4A) and again abrogated by pretreatment with geminin (Figure 4B). Similarly, endogenous PrimPol in normal human fibroblasts partitioned to the detergent-insoluble chromatin-enriched fraction following aphidicolin treatment (Figures S4A and S4B). Together, these data indicate PrimPol loads onto chromatin in a replication-dependent manner, and this chromatin association is even more apparent following replication fork stalling. Finally, we generated *PrimPol*^*−/−*^ mice and subsequently mouse embryonic fibroblasts (MEFs) lacking expression of the *PrimPol* gene (Figures S4C–S4F) and analyzed metaphase chromosome spreads. We observed an increase of metaphase aberrations in MEFs lacking PrimPol, in particular chromatid breaks (Figure 4C), which are indicative of lesions arising during S phase. Further, following a prolonged treatment with low-dose aphidicolin, the percentage of metaphase aberrations increased substantially (Figure 4C). This supports a role for PrimPol in protecting replication forks from replication stress, which would be consistent with the aphidicolin- and HU-induced chromatin binding of PrimPol observed previously. Whether this role is reinitiation of DNA synthesis de novo or akin to the role of Y-family polymerases during replication stress (de Feraudy et al., 2007, Godoy et al., 2006, Bétous et al., 2013) remains to be established.

## Discussion

Here, we extend the cellular role of AEPs, previously shown to function in initiation of DNA replication (Frick and Richardson, 2001) and prokaryotic DNA break repair processes (Della et al., 2004, Pitcher et al., 2007), by uncovering a function in bypassing UV lesions during chromosomal DNA replication in eukaryotic cells. There are at least two possible mechanisms of PrimPol-mediated fork progression (Figure 4D). With regards to damaged DNA templates, such as those containing UV lesions, PrimPol is a flexible DNA polymerase and thus can extend from a blocked primer terminus by directly synthesizing across the lesion. PrimPol is also a primase and therefore has the capacity to initiate DNA synthesis de novo downstream of the lesion following fork stalling, producing a ssDNA gap in the daughter strand. Presumably, the latter process would be restricted to lesions on the leading strand, as discontinuous synthesis of Okazaki fragments on the lagging strand is inherently tolerant of DNA lesions (Svoboda and Vos, 1995, Pagès and Fuchs, 2003). Evidence of repriming downstream of UV photoproducts exists in mammalian cells (Lehmann, 1972, Elvers et al., 2011), and most convincingly, in budding yeast, ssDNA gaps in the leading strand have been visualized behind replication forks in UV-irradiated NER-deficient cells (Lopes et al., 2006). However, as PrimPol is absent from the majority of fungi, including budding yeast (Iyer et al., 2005), this repriming is likely dependent upon the replicative DNA primase (Prim1) of the Pol α complex. Similarly, in *E. coli*, in which leading-strand repriming has been mechanistically described in vitro, the replicative DnaG primase is required for this process (Heller and Marians, 2006, Yeeles and Marians, 2011). By extrapolation, this suggests that if PrimPol reprimes downstream of leading strand lesions, it may function redundantly with the Pol α complex. Studies in *Xenopus* have demonstrated the Pol α complex is responsible for the bulk of primer synthesis at stalled replication forks (Michael et al., 2000, Van et al., 2010), although this may reflect the abundance of primers on the lagging versus leading strand. In support of PrimPol-mediated TLS, a number of studies have reported a common misinsertion of dT opposite the 3′ T of a 6-4 PP in vivo, with the polymerase involved remaining unidentified (Hirota et al., 2010, Szüts et al., 2008), suggesting that PrimPol may be responsible for incorporation opposite a subset of these lesions. PrimPol’s ability to catalyze TLS opposite 6-4PPs could be particularly important, as they pose a significant block to replicases. Interestingly, the nuclear replicase Pol δ has been reported to incorporate two dA residues opposite a T-T CPD in vitro under certain circumstances (Narita et al., 2010, O’Day et al., 1992), and therefore PrimPol may function in concert with the cellular replicases to bypass CPDs. We also establish that PrimPol functions during unperturbed chromosomal DNA replication. PrimPol may be required for the bypass of naturally occurring DNA lesions or structures either by repriming or as previously described for TLS polymerases (Bergoglio et al., 2013). Regardless of the mechanism of PrimPol-assisted fork progression, it is evident that this AEP is an important player at stalled replication forks in eukaryotic cells.

## Experimental Procedures

### Protein Preparation and Activity Assays

Human *PrimPol* cDNA was subcloned into pET28a (NdeI, BamHI), and the catalytic null mutant was obtained by site-directed mutagenesis. Both proteins were expressed in *E. coli* BL21 and purified with Ni^2+^-NTA (QIAGEN) followed by heparin and size-exclusion (GE Healthcare) chromatography columns. Primer extension assays were performed with 5′ Hexachlorofluorescein-labeled DNA primers (ATDbio). De novo DNA synthesis in the primase assay was detected by FAM-6-dATP (Jena-Biosciences) labeling of reaction products. Further details of protein preparation, primer extension assays, and the fluorescent primase assay are in the Supplemental Experimental Procedures.

### Mammalian Cell Culture and RNA Interference

143B, HEK293 Flp-In T-REx (Invitrogen), and MEFs were cultured in DMEM supplemented with 10% fetal calf serum (FCS), and SV40-transformed MRC5, XP30RO, and XPA cells were cultured in MEM supplemented with 15% FCS. All cells were grown in 1% L-glutamine and 1% PenStrep. Generation of the HEK293 Flp-In T-REx stable cell lines is detailed in the Supplemental Experimental Procedures. Transfection of 10 nM PrimPol siRNA (5'-GAGGAAACCGUUGUCCUCAGUGUAU-3', 5'-AUACACUGAGGACAACGGUUUCCUC-3') was performed using Lipofectamine 2000 (for 143b cells) or Lipofectamine RNAiMAX (Invitrogen) according to manufacturer recommendations.

### Immunofluorescent Analysis and Cellular Fractionation

For immunofluorescent analysis, rabbit anti-PrimPol antibody (generated in house) was diluted 1:200, anti-RPA2 (Cell Signaling) 1:200, and anti-RAD51 (Abcam) 1:400. Cellular fractionation protocol was adapted from Kannouche et al., 2004. Further details are provided in Supplemental Experimental Procedures.

### Clonogenic Survival Assay

MRC5, XP30RO, and XPA cells were either mock or PrimPol siRNA transfected, both at seeding and 24 hr after. At 72 hr dilutions of the transfected cells were plated in triplicate on 10 cm dishes. The next day the media were removed and cells were washed with PBS and then UV-C irradiated in the presence or absence of caffeine (0.38 mM). After irradiation media were replaced and colonies were allowed to grow over 10 days, before fixing with 100% ethanol and staining with methylene blue to allow counting.

### Generation of PrimPol Knockout Avian DT40 Cells and DNA Replication/Repair Assays

Avian *PrimPol* gene was disrupted with histidinol- and puromycin-targeted cassettes following procedures described previously (Sonoda et al., 1998). Human *PrimPol* cDNA was subcloned into TET inducible vector (adapted from Clontech) containing luciferase gene reporter for clone selection. Further details can be found in Supplemental Experimental Procedures. DNA fiber analysis and the postreplication repair assay were performed as previously described (Edmunds et al., 2008). Postreplication repair protocol was adapted to measure total DNA replication rate following UV-C, by prelabeling cells with ^14^C thymidine over two doubling times to normalize the amount of total DNA.

### *Xenopus* Egg Extract Preparation and Assays

Demembranated sperm nuclei and interphase *Xenopus* egg extracts were prepared as described (Murray et al., 1991, Kubota and Takisawa, 1993). Further details are available in Supplemental Experimental Procedures.

### Generation of PrimPol Knockout MEFS and Metaphase Chromosome Preparations

The targeting of the *PrimPol* locus in mouse embryonic stem cells, generation of the *PrimPol* knockout mouse, and primary mouse fibroblasts lacking the *PrimPol* gene are detailed in Figure S4. Mitotic MEF populations were enriched by colcemid (2 × 10^−7^ M) treatment for 1–2 hr and, following harvesting, were osmotically swollen, fixed, and placed on glass slides before staining with 5% Giemsa and microscopic analysis. See Supplemental Experimental Procedures for further detail.

## Supplemental Information

Document S1. Figures S1–S4, Tables S1–S3, and Supplemental Experimental Procedures
