# Supplementary material for: PrimPol Bypasses UV Photoproducts during Eukaryotic Chromosomal DNA Replication
Source: Mol Cell. 2013 Nov 21;52(4):566–73. doi: 10.1016/j.molcel.2013.10.035 (PMC4228047; doi:10.1016/j.molcel.2013.10.035)
Supplement: Document S1. Figures S1–S4, Tables S1–S3, and Supplemental Experimental Procedures [file mmc1.pdf]

Molecular Cell, Volume *52*

Supplemental Information

PrimPol Bypasses UV Photoproducts during Eukaryotic Chromosomal DNA Replication

Julie Bianchi, Sean G. Rudd, Stanislaw K. Jozwiakowski, Laura J. Bailey, Violetta Soura, Elaine Taylor, Irena Stevanovic, Andrew J. Green, Travis H. Stracker, Howard D. Lindsay, and Aidan J. Doherty

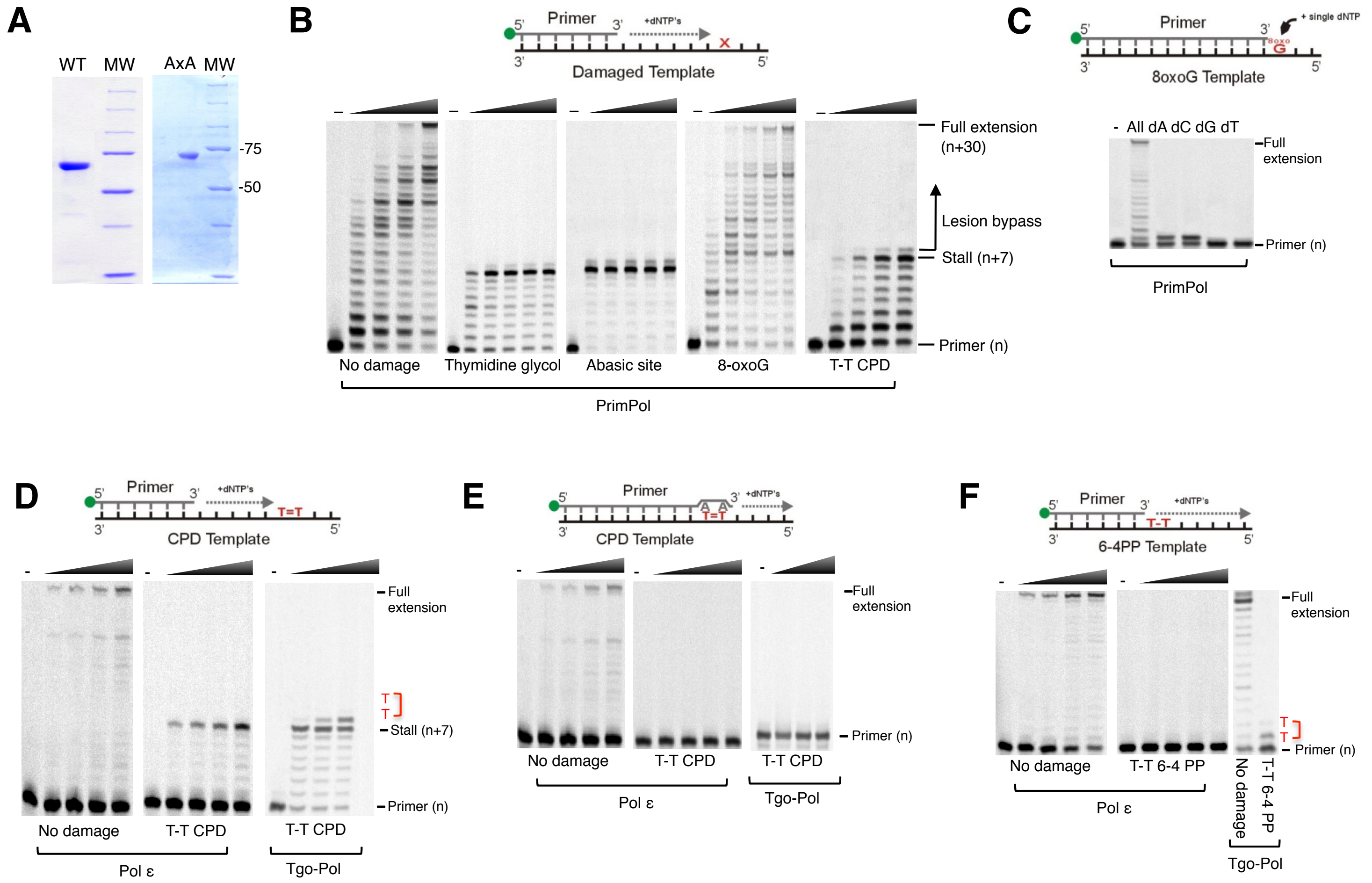
A
WT MW
AxA MW
-75
-50
B
Primer
+dNTP's
Damaged Template
Full extension (n+30)
Lesion bypass
Stall (n+7)
Primer (n)
No damage
Thymidine glycol
Abasic site
8-oxoG
T-T CPD
PrimPol
C
Primer
+ single dNTP
8oxoG Template
- All dA dC dG dT
Full extension
Primer (n)
PrimPol
D
Primer
+dNTP's
CPD Template
Full extension
Stall (n+7)
Primer (n)
No damage
T-T CPD
T-T CPD
Pol ε
Tgo-Pol
E
Primer
+dNTP's
CPD Template
Full extension
Primer (n)
No damage
T-T CPD
T-T CPD
Pol ε
Tgo-Pol
F
Primer
+dNTP's
6-4PP Template
Full extension
Primer (n)
No damage
T-T 6-4 PP
No damage
T-T 6-4 PP
Pol ε
Tgo-Pol

Figure S1

Figure S1. Primer extension assays with human PrimPol, human Pol ε, and archaeal replicase Tgo-Pol (linked to Figure 1)

(**A**) SDS PAGE analysis of wild-type (WT) His-tagged human PrimPol and catalytic mutant (AxA).

(**B**) DNA synthesis by PrimPol on DNA templates containing a thymidine glycol lesion, abasic site, 8-oxo-Guanine (8-oxoG), or T-T *cys-sin* cyclobutane pyrimidine dimer (CPD), was compared to DNA synthesis on an undamaged template using primer extension assays. Primer-template substrate (20 nM) and dNTPs (200 µM) were incubated with or without (–) WT PrimPol (50 nM) at 37°C for increasing times (2, 5, 10, 15 minutes, indicated by the black triangle). Thymidine glycol, abasic site, and 8-oxoG were positioned at bases +8 of the template relative to 3' terminus of primer, whilst the CPD was positioned at bases +8 and +9, to test read-through by PrimPol.

(**C**) DNA synthesis by PrimPol on a DNA template containing an 8-oxoG lesion. Primer extension performed as in (B) except individual dNTPs were added for a single 30 minute time point. 8-oxoG is at base +1 of the template relative to the 3' terminus of the primer to test single nucleotide incorporation opposite the lesion.

(**D** – **F**) DNA synthesis by human Pol ε and archaeal replicase from *Thermococcus gorgonarius* (Tgo-Pol exo-) on DNA templates containing a T-T CPD (**D** and **E**) or a T-T pyrimidine (6-4) pyrimidone photoproduct (6-4 PP) (**F**) was compared to DNA synthesis on undamaged templates using primer extension assays. Human Pol ε (100 nM) was incubated with the indicated primer-template substrate (20 nM) and dNTPs (200 µM) in reaction buffer for increasing times (2, 5, 10, 20 minutes, indicated by black triangle) at 37°C. For Tgo-Pol reactions, 50 nM enzyme was used and the reaction incubated for a single 30 minutes time point or increasing times (30, 60, 120 seconds, black triangle). No enzyme controls (–) were performed in similar conditions. CPD is at bases +8 and +9 of template relative to 3' terminus of primer to test read-through (D), or annealed opposite two 3' terminal dA residues thereby testing polymerase extension opposite the lesion (E). 6-4 PP is at bases +1 and +2 of template relative to 3' terminus of primer to test read-through (F).

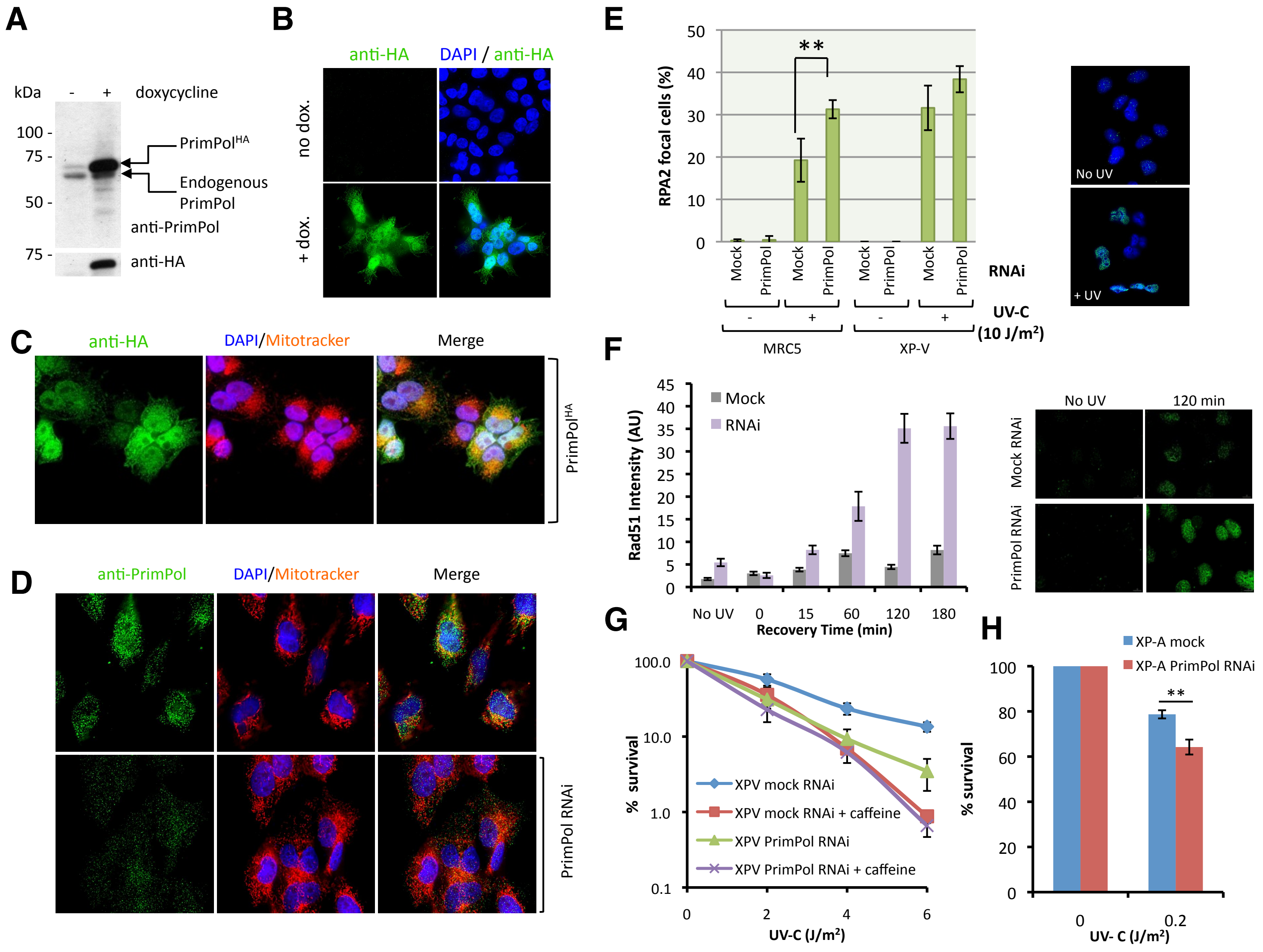
A
B
E
kDa
-
+
doxycycline
100 -
75 -
50 -
75 -
PrimPol^HA
Endogenous PrimPol
anti-PrimPol
anti-HA
anti-HA
DAPI / anti-HA
no dox.
+ dox.
RPA2 focal cells (%)
**
Mock
PrimPol
RNAi
UV-C (10 J/m²)
MRC5
XP-V
No UV
+ UV
C
anti-HA
DAPI/Mitotracker
Merge
PrimPol^HA
F
Rad51 Intensity (AU)
Mock
RNAi
No UV
120 min
Mock RNAi
PrimPol RNAi
Recovery Time (min)
D
anti-PrimPol
DAPI/Mitotracker
Merge
PrimPol RNAi
G
% survival
XPV mock RNAi
XPV mock RNAi + caffeine
XPV PrimPol RNAi
XPV PrimPol RNAi + caffeine
UV-C (J/m²)
H
XP-A mock
XP-A PrimPol RNAi
% survival
UV- C (J/m²)

Figure S2

Figure S2. PrimPol sub-cellular localisation and RNAi phenotype analysis in cultured human cells (linked to Figure 2)

(**A** - **C**) Flp-In T-REx-293 cells engineered for inducible expression of recombinant PrimPol fused to a C-terminal HA epitope ($PrimPol^{HA}$) were grown in the absence or presence of 10 ng/ml doxycycline for 18 hours before analysis. Cell lysates were prepared and analysed by Western blot with an anti-PrimPol and an anti-HA antibody (**A**). Fixed cells were subjected to immunofluorescent analysis with an anti-HA antibody (green) and DAPI counterstaining (blue) (**B**). Prior to fixing and immunofluorescent staining with anti-PrimPol (green) and counterstainig with DAPI (blue), cells were incubated with Mitotracker Deep Red (red) to stain mitochondria (**C**).

(**D**) Immunofluorescent detection of endogenous PrimPol. 143B cells were either mock or PrimPol RNAi treated and 48 hours after, subjected to immunofluorescent analysis with an anti-PrimPol antibody (green). Cells were counterstained with Mitotracker Deep Red to visualize mitochondria (red) and DAPI to visualize nuclei (blue).

(**E**) Normal (MRC5) and XP-V (XP30RO) fibroblasts were either mock or PrimPol RNAi treated and at 72 hours seeded on glass coverslips. The next day cells were either mock or UV-C (10 J/m^2) irradiated and following a 6 hour recovery, were Triton X-100 (0.5%) extracted and subjected to immunofluorescent analysis with anti-RPA2 and DAPI counterstaining. The proportion of cells with focal RPA2 (> 20 foci per nuclei) were determined, at least 200 cells were counted from each sample, error bars denote SD of 3 experiments. Significance was determined with the two-tailed T-test (**< 0.01). Representative images are shown on the right panel.

(**F**) XP-V cells were either mock or PrimPol RNAi treated then either mock or UV-C (3 J/m^2) irradiated; following different recovery times, cells were Triton X-100 (0.1%) extracted and subjected to immunofluorescent analysis with Rad51 antibody and DAPI counterstaining. Intensity of the signal was measured by Image J, error bars denote SD of 2 experiments. Representative images are shown on the right panel.

(**G**) XP-V cells were mock or PrimPol RNAi treated. At 24 hours they were seeded on 9cm dishes in duplicate and allowed to attach. Cells were then treated with 0-6 J/m^2 UV-C with or without the addition of 0.38 mM caffeine. Colonies were allowed to form over 10 days when cells were fixed and stained with methylene blue. Error bars show SD of 3 experiments.

(**H**) Role of PrimPol in NER. XP-A cells were mock or PrimPol RNAi treated. Following RNAi cells were seeded to replicate 9cm plates and allowed to attach. Cells were then treated with 0 or 0.2 J/m^2 UV-C and

colonies were allowed to form over approximately 10 days. Colonies were stained with methylene blue and counted to compare survival. Error bars represent SD of the mean of 3 experiments, significance was determined using the T-test ($p = 0.006$).

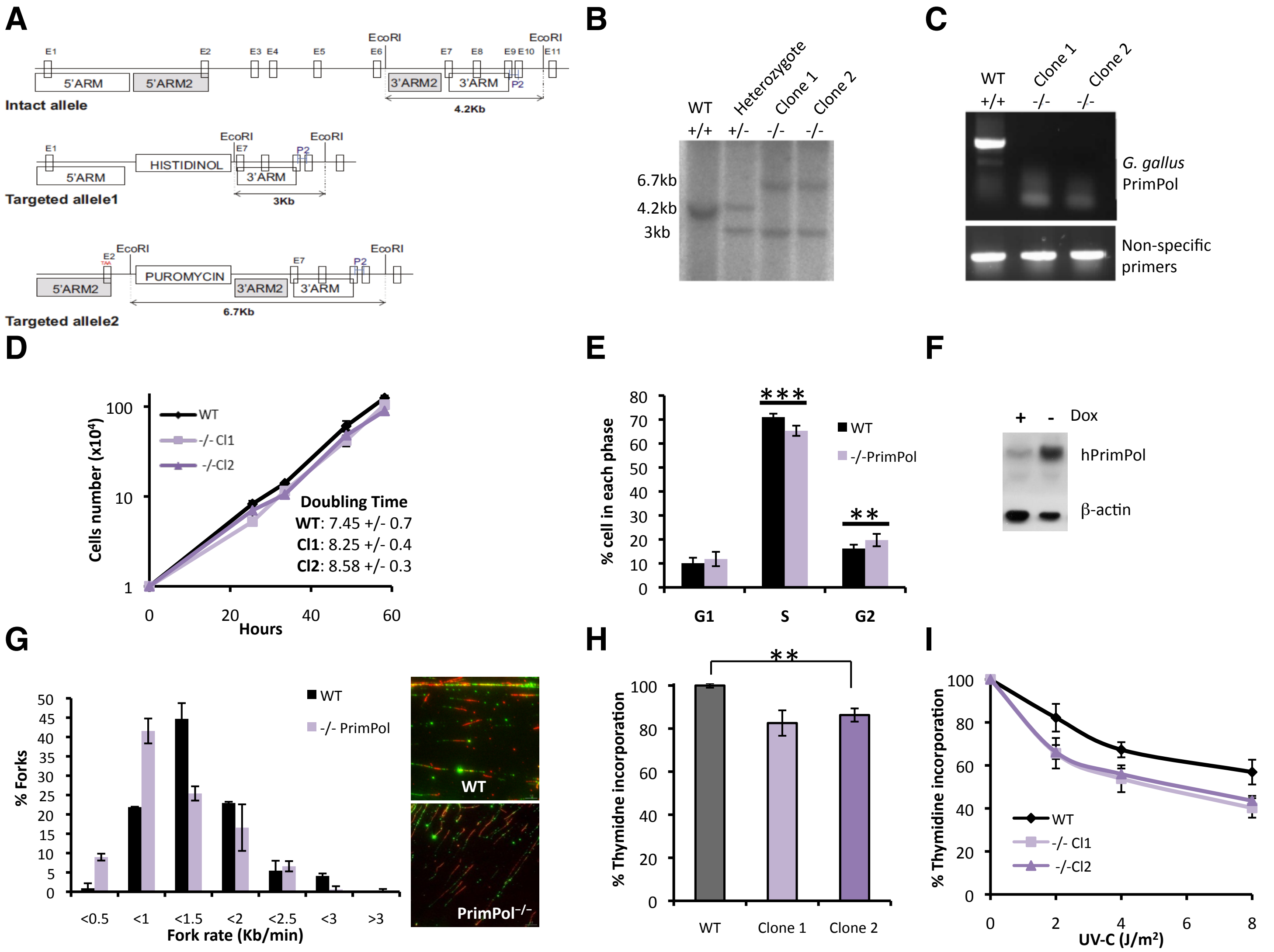
A
EcoRI
EcoRI
E1
E2
E3 E4
E5
E6
E7
E8
E9 E10
E11
5'ARM
5'ARM2
3'ARM2
3'ARM
P2
Intact allele
4.2Kb
EcoRI
EcoRI
E1
E7
P2
5'ARM
HISTIDINOL
3'ARM
Targeted allele1
3Kb
EcoRI
EcoRI
E2
E7
P2
5'ARM2
PUROMYCIN
3'ARM2
3'ARM
Targeted allele2
6.7Kb
B
WT +/+
Heterozygote +/-
Clone 1 -/-
Clone 2 -/-
6.7kb
4.2kb
3kb
C
WT +/+
Clone 1 -/-
Clone 2 -/-
G. gallus PrimPol
Non-specific primers
D
Cells number (x10⁴)
Hours
WT
-/- Cl1
-/-Cl2
Doubling Time
WT: 7.45 +/- 0.7
Cl1: 8.25 +/- 0.4
Cl2: 8.58 +/- 0.3
E
% cell in each phase
WT
-/-PrimPol
G1
S
G2
F
+ - Dox
hPrimPol
β-actin
G
% Forks
Fork rate (Kb/min)
WT
-/- PrimPol
<0.5
<1
<1.5
<2
<2.5
<3
>3
WT
PrimPol-/-
H
% Thymidine incorporation
WT
Clone 1
Clone 2
I
% Thymidine incorporation
UV-C (J/m²)
WT
-/- Cl1
-/-Cl2

Figure S3

Figure S3. Generation and characterisation of PrimPol knockout DT40 cell lines (linked to Figure 3)

(**A**) Illustration of Southern blot strategy used to disrupt *G. gallus PrimPol* gene; exons (square boxes), EcoRI restriction sites and Southern blot probe (P2) are depicted along with expecting band size (under horizontal arrows) for Southern blot analysis. Both disruption cassettes (histidinol and puromycin) are flanked by two distinct sets of recombination arms (white or grey boxes) in order to specifically target the wild-type allele remaining in heterozygote clones WT/HIS.

(**B**) Southern blot of genomic DNA of WT DT40 cells (+/+) and clones obtained after *PrimPol G. gallus* gene disruption showing the loss of both wild-type alleles in clone 1 and 2 (–/–) or only one allele remaining in the heterozygote clone (+/–).

(**C**) RT-PCR performed on RNA extracted from WT and both KO clones, using *G. gallus PrimPol* specific primers (upper gel) or unspecific *G. gallus* primers (lower gel) as PCR control.

(**D**) Growth curves of WT (black line) and *PrimPol*$^{-/-}$ (light and dark purple) DT40 cells were assessed by counting duplicate cultures of each population over 7 generations (56 hours), using a haemocytometer with trypan blue dye exclusion staining. Doubling time for each population was determined using an exponential regression algorithm; average and SD were calculated from 3 independent experiments.

(**E**) Cell cycle profile of WT DT40 (black) or average of both *PrimPol*$^{-/-}$ clones (purple) was determined using flow cytometry after BrdU incorporation. Error bars denote SD of 5 experiments, and significance was determined with the two-tailed T-test (***<0.001, **<0.01).

(**F**) Western blot analysis of *PrimPol*$^{-/-}$ clone expressing human PrimPol under a tetracycline (tet off) promoter; doxycycline was added at 1 mg/ml for 24 hours; anti-PrimPol and anti beta-actin antibodies were used.

(**G**) Distribution of replication fork speeds in unperturbed wild-type (WT) and *PrimPol*$^{-/-}$ DT40 cells was determined by DNA fiber analysis. Error bars denote SD of 2 experiments with > 100 DNA fibers analyzed each time, with two *PrimPol*$^{-/-}$ cell lines used.

(**H**) WT (black line) and both *PrimPol*$^{-/-}$ (light and dark purple lines) DT40 cells were pulse-labeled for 20 minutes with ^{3}H-thymidine following UV-C irradiation. Incorporation rate was calculated and normalised against undamaged cells using ^{14}C-thymidine overnight pre-labeling for total DNA normalisation. Error bars represent SD of 3 independent experiments.

(**I**) WT (grey) and *PrimPol*$^{-/-}$ (light and dark purple) DT40 cells were first labeled with ^{14}C-thymidine for 16 hours and then pulse-labeled for 20 minutes with ^{3}H-thymidine. Incorporation rate was calculated after normalisation with ^{14}C signal for total DNA content. Error bars represent SD of 3 independent experiments.

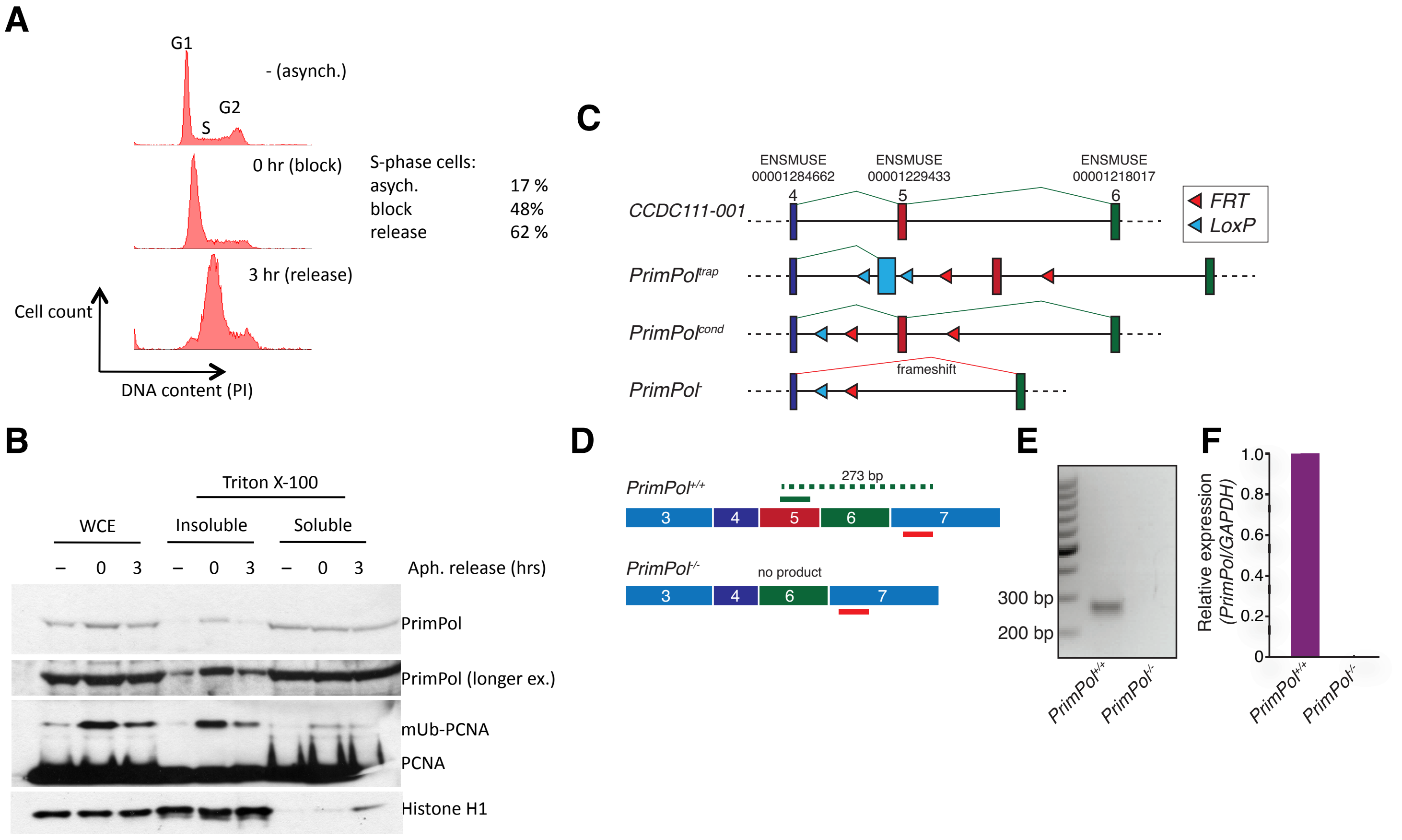
A
G1
- (asynch.)
G2
S
0 hr (block)
S-phase cells:
asych. 17 %
block 48%
release 62 %
3 hr (release)
Cell count
DNA content (PI)
B
Triton X-100
WCE
Insoluble
Soluble
– 0 3 – 0 3 – 0 3
Aph. release (hrs)
PrimPol
PrimPol (longer ex.)
mUb-PCNA
PCNA
Histone H1
C
ENSMUSE 00001284662
ENSMUSE 00001229433
ENSMUSE 00001218017
4
5
6
CCDC111-001
FRT
LoxP
PrimPol trap
PrimPol cond
frameshift
PrimPol-
D
PrimPol+/+
273 bp
3 4 5 6 7
PrimPol-/-
no product
3 4 6 7
E
300 bp
200 bp
PrimPol+/+
PrimPol-/-
F
Relative expression
(PrimPol/GAPDH)
1.0
0.8
0.6
0.4
0.2
0
PrimPol+/+
PrimPol-/-

Figure S4

Figure S4. PrimPol chromatin-association following aphidicolin treatment and generation of PrimPol knockout MEFs (linked to Figure 4)

(**A**) SV40-transformed normal (MRC5) fibroblasts were grown in the presence or absence of 2 µg/ml aphidicolin for 16 hours, and then the aphidicolin was removed and cells allowed to grow for a further 3 hours before analysis. Cells were fixed, stained with PI, and analysed by flow cytometry to determine the DNA content and the proportion of S-phase cells.

(**B**) PrimPol becomes insoluble following prolonged aphidicolin treatment, suggesting chromatin binding. MRC5 fibroblasts were either mock (-) or aphidicolin treated (2 µg/ml, ~16 hours) and allowed to recover for the time indicated before being separated into Triton X-100 soluble and insoluble fractions and subjected to Western blot analysis with antibodies indicated. Histone H1 was used to detect chromatin, PCNA and the slower migrating mono-ubiquitylated (Ub)-PCNA indicates stalled replication forks.

(**C**) Schematic of modified murine *PrimPol* locus (*CCDC111*). Dark blue and green boxes represent exons, red box the targeted exon 5 (contains the entire AEP I signature motif), light blue box the genetrap cassette, light blue triangles FRT sites, red triangles LoxP sites and green lines predicted splicing patterns confirmed by analysis of cDNA.

(**D**) Real time PCR strategy for the assessment of relative mRNA levels in MEFs.

(**E**) Agarose gel electrophoresis of PCR products from PrimPol primers and cDNA of the indicate genotype.

(**F**) Real time PCR analysis of PrimPol mRNA levels in wild type and *PrimPol*$^{-/-}$ animals.

Table S1. Primer-template substrates (related to Experimental Procedures)

Figure	Primer (5’→3’)	Template (5’→3’)
1C	TGTCGTCTGTTCGGTCGTTC	CGCGCAGGGCGCACAACAGCCTTGAAGACCGAACGACCGAACAGACGACA
1D	TGTCGTCTGTTCGGTCGTTCGGTCTTCAA	CGCGCAGGGCGCACAACAGCCTTGAAGACCGAACGACCGAACAGACGACA or<br>CGCGCAGGGCGCACAACAGCC(T=T)GAAGACCGAACGACCGAACAGACGACA
1E	CACTGACTGTATGATG	CTCGTCAGCATCTTCATCATACAGTCAGTG or<br>CTCGTCAGCATC(T/T)CATCATACAGTCAGTG
1F	CACTGACTGTATGATG	CTCGTCAGCATCTTCATCATACAGTCAGTG or<br>CTCGTCAGCATC(T/T)CATCATACAGTCAGTG
1F	CACTGACTGTATGATGT	CTCGTCAGCATC(T/T)CATCATACAGTCAGTG
1G	CACTGACTGTATGATGT	CTCGTCAGCATCTTCATCATACAGTCAGTG or<br>CTCGTCAGCATC(T/T)CATCATACAGTCAGTG
S1B	TGTCGTCTGTTCGGTCGTTC	CGCGCAGGGCGCACAACAGCCTTGAAGACCGAACGACCGAACAGACGACA or<br>CGCGCAGGGCGCACAACAGCC(Tg)TGAAGACCGAACGACCGAACAGACGACA or<br>CGCGCAGGGCGCACAACAGCC(AP)TGAAGACCGAACGACCGAACAGACGACA or<br>CGCGCAGGGCGCACAACAGCC(oxoG)TGAAGACCGAACGACCGAACAGACGACA or<br>CGCGCAGGGCGCACAACAGCC(T=T)GAAGACCGAACGACCGAACAGACGACA
S1C	TGTCGTCTGTTCGGTCGTTCGGTCTTCA	CGCGCAGGGCGCACAACAGCC(oxoG)TGAAGACCGAACGACCGAACAGACGACA
S1D	TGTCGTCTGTTCGGTCGTTC	CGCGCAGGGCGCACAACAGCCTTGAAGACCGAACGACCGAACAGACGACA or<br>CGCGCAGGGCGCACAACAGCC(T=T)GAAGACCGAACGACCGAACAGACGACA
S1E	TGTCGTCTGTTCGGTCGTTCGGTCTTCAA	CGCGCAGGGCGCACAACAGCCTTGAAGACCGAACGACCGAACAGACGACA or<br>CGCGCAGGGCGCACAACAGCC(T=T)GAAGACCGAACGACCGAACAGACGACA
S1F	CACTGACTGTATGATG	CTCGTCAGCATCTTCATCATACAGTCAGTG or<br>CTCGTCAGCATC(T/T)CATCATACAGTCAGTG

DNA lesions are indicated in red text: T=T, thymine-thymine *cys-sin* cyclobutane pyrimidine dimer (T-T CPD); T/T, thymine-thymine pyrimidine 6-4 pyrimidone photoproduct (T-T 6-4 PP); Tg, thymidine glycol; AP, abasic site; oxoG, 8-oxo-Guanine.

Table S2. DT40 PrimPol knockout primers (linked to Experimental Procedures)

Histidinol upstream ARM primer FOR	5'-GGGGACAACTTTGTATAGAAAAGTTGGGCGGCAGCGCGCGGGCCGCGGAGAGG-3'
Histidinol upstream ARM primer REV	5'-GGGGACTGCTTTTTTGTACAAACTTGTATACATTTAAATATCTTTGTGGATC-3'
Histidinol downstream ARM primer FOR	5'-GGGGACAGCTTTCTTGTACAAAGTGGTGAGAGGGGAGCTGAAAGCAGTTT-3'
Histidinol downstream ARM primer REV	5'-GGGGACAACTTTGTATAATAAAGTTGCCATGGGCTCTGTTTAAACAAACAAGC-3'
Puromycin upstream ARM primer FOR	5'-GGGGACAACTTTGTATAGAAAAGTTGTTAAAGGTTTGAGTGATTCTGAATATAGCC-3'
Puromycin upstream ARM primer REV	5'-GGGGACTGCTTTTTTGTACAAACTTGTTACTCCTTGCAGGTTTTCACAAATC-3'
Puromycin downstream ARM primer FOR	5'-GGGGACAGCTTTCTTGTACAAAGTGGAACCAAATACAGTCCATGCTGAAAC-3'
Puromycin downstream ARM primer REV	5'-GGGGACAACTTTGTATAATAAAGTTGGTGGCCAACTAAGACTATGCAAAC-3'

Table S3. Southern blot probe and RT-PCR primers (linked to Experimental Procedures)

Southern blot probe, primer FOR	5'-ACGGAGTGCAAGGAGGTAAC-3'
Southern blot probe, primer REV	5'-GACTGGATCGTGGCACTTC-3'
G. gallus PrimPol cDNA, primer FOR	5'-ATGAAGAGAAAATGGGAAGAAAGAGTGAAGAAAGTTG -3'
G. gallus PrimPol cDNA, primer REV	5'-AGCCTTTGGAAGCATCTTCGACT-3'
G.gallus Bora cDNA, primer FOR	5'- ATGGGCGATACAGAAGAAGCCCAAATGCAG-3'
G.gallus Bora cDNA, primer REV	5'-CTCGAGAGGAGAAGGAACTATGAGACTCTTTGTGAAAACTCC-3'

SUPPLEMENTARY EXPERIMENTAL PROCEDURES

Purification of recombinant proteins

Human PrimPol cDNA was sub-cloned into pET28a (Novagen) using NdeI and BamHI restriction sites to generate a 6-histidine N-terminal tagged recombinant protein. Catalytic null mutant AxA (D114A and E116A) was obtained by site directed mutagenesis PCR of this construct using forward 5'-AGCTTTATTTTGCTTTGGCATTTAACAAACC-3' and reverse 5'-GGTTTGTTAAATGCCAAAGCAAAATAAAGCT-3' primers. Both proteins were expressed using Bl21 *E. coli* strain following overnight induction at 16°C by adding 0.4 mM IPTG and purified with Ni^{2+}-NTA (Qiagen) followed by heparin and size-exclusion (GE Healthcare) chromatography columns.

Archaeal family-B DNA polymerase from *Thermococcus gorgonarius* (Tgo PolB exo^{-}) used in control primer extension reactions on synthetic substrates containing UV induced DNA lesions was purified as described previously (Evans et al., 2000). The human DNA polymerase ε holoenzyme was kindly provided by Professor Juhani Syväoja (University of Eastern Finland, Finland).

Fluorescent primer template based assays

The HPLC grade DNA oligomers used to prepare synthetic primer-template substrates were purchased from ATDbio (Southampton, UK). The DNA oligomer containing pyrimidine-pyrimidone (6-4) photoproduct was kindly provided by Professor Shigenori Iwai (Osaka University, Japan). Primers contained a 5' Hex fluorophore label. Primer and template sequences are detailed in supplementary Table 1.

All the primer extension assays were performed at 37ºC as described previously (Jozwiakowski and Connolly, 2011). The typical primer extension reaction was performed in 20 µl volume containing 10 mM Bis-Tris-Propane-HCl, pH 7,0, 10 mM NaCl, 10 mM $MgCl_2$, 1 mM DTT, 20 nM primer-template substrate, 200 µM dNTP's (Roche) with 50 nM recombinant human PrimPol. The products of the primer-template extension reactions were monitored over time course of 2, 5, 10, 15 minutes. All single incorporation assays were performed in the presence of single dNTP and reactions were quenched after 30 minutes incubation.

Control primer extension with Tgo-PolB exo- were performed at 50ºC in 20 µl volume containing 20 mM Tris-HCl, pH 8,5, 20 mM NaCl, 2 mM MgSO4, 20 nM primer-template substrate, 100 µM dNTP's (Roche)

with 50 nM recombinant Tgo PolB exo-. Control primer extension with human Pol ε were performed in 20 µl volume containing 10 mM Bis-Tris-Propane-HCl, pH 7,0, 10 mM NaCl, 10 mM $MgCl_2$, 1 mM DTT, 20 nM primer-template substrate, 200 µM dNTP's (Roche) with 100 nM human DNA polymerase ε. The products of the primer-template extension reactions were monitored over time course of 2, 5, 10, 20 minutes.

Fluorescent primase assay

The non-radioactive primase assay was performed in three steps. Typically detection of primase activity was started from incubation of 1 µM of the tested enzyme or 2 U of klenow-Taq (negatve control for the assay; purified as described in Engelke et al., 2000) in 20 µl reaction volume containing 500 nM ssDNA synthetic template (dAx60, dCx60, dGx60, dTx60) with a biotin modification at the 5' end, 500 µM rNTPs (Invitrogen) or 500 µM dNTPs (Roche), 10 mM Bis-Tris-Propane-HCl pH7, 10 mM $MgCl_2$, 50 mM NaCl. Primer synthesis was carried out for 2 hours at 37°C then reaction supplemented with 0.2 U of kTaq and 15 µM FAM-6-dATP (Jena-Biosciences) and incubated at 37°C for 45 minutes to allow fluorescent labeling of *de novo* synthesised primers. The primer synthesis/labeling enzymatic reactions were terminated by adding 450 µl of binding-washing (B-W) buffer (10 mM Tris-HCl 8.0, 500 mM NaCL, 10mM EDTA). The quenched reactions were added to ~30 µl of streptavidin coated beads (500 µl total volume) and mixed on a spinning wheel for 1 hour at 4°C. After capturing the ssDNA templates, the suspension were spun down briefly to sediment the beads. The supernatant was removed and the beads were washed three times with 1 ml volumes of B-W buffer. The beads were then suspended in 20 µl of 8 M UREA 10 mM EDTA and boiled for 3 minutes in order to liberate primers. The 20 µl samples were spun down briefly, loaded on 15% polyacrylamide / 7M urea gel and resolved for 105 minutes at 17 watts. After electrophoresis gels were scanned for fluorescent signal and products of reaction/labeling of *de novo* synthesised primers were visualised.

Generation of a stable inducible cell line

Flp-In™ T-REx™ HEK-293 cells (Invitrogen) were used to make a stable cell line with inducible expression of epitope tagged PrimPol. Prior to transfection, cells were grown in 100 µg/ml Zeocin (Invitrogen) and 15 µg/ml Blasticidine (Invitrogen). Cells were seeded in a 6-well plate 24 hours after transfection with pcDNA5/FRT/TO plasmid encoding PrimPol-FlagStrep or PrimPol-HA, and pOG44 plasmid with Lipofectamine 2000 according to the manufacturer's instructions. 48 hours after, cells were split into 10 cm dishes and 72 hours after 15 µg/ml Blasticidine and 100 µg/ml Hygromycin (Invitrogen) added. Selective medium was replaced every 3-4 days until clones appeared visible and stocks made.

Immunofluorescent analysis

Cells were grown on coverslips, which for HEK-293 Flp-In^{TM} T-REx^{TM} cells were poly-L-lysine coated. Following siRNA transfection and/or DNA damaging treatments and recovery time, cells were either fixed directly in 3% paraformaldehyde (in PBS) for 15 minutes or pre-extracted by washing in 0.5% Triton X-100 in PBS before fixing. Cells were permeabilised with 0.2% Triton X-100 in PBS for 10 minutes, blocked with 3% BSA in PBS before immuno-staining steps. Coverslips were mounted on slides with Prolong Gold anti-fade (Invitrogen). Slides were analysed on a widefield Deltavision microscope for images, and a Nikon E400 for counting.

Antibodies used were anti-PrimPol 1:200 (in house), anti-RPA2 1:200 (Cell Signalling), anti-RAD51 1:400 (Abcam). Secondary antibodies were Alexa Fluor 488 goat anti-rabbit 1:2000, and Alexa Fluor 594 goat anti-mouse 1:2000. Cells were incubated with 250 nM Mitotracker Deep Red (final concentration) (Invitrogen) for 30 minutes prior to fixation. Immunolabeling of DNA fibers was performed with anti-rat BrdU (abcam) 1:1000, anti-mouse BrdU (Becton Dickinson) 1:500 and secondary Alexa Fluor 488-labeled anti-rat and Alexa Fluor 594-labeled anti-mouse (Invitrogen) both 1:250.

Cellular fractionation

Cellular fractionation protocol was modified from (Kannouche et al., 2004; Zlatanou et al., 2011). Cell pellet was resuspended in cytoskeletal (CSK) buffer (100 mM NaCl, 300 mM sucrose, 3 mM MgCl2, 10 mM Pipes (pH 6.8), 1 mM EGTA, 0.2% Triton X- 100) supplemented with protease and phosphatase inhibitors (Roche), and incubated on ice for 5 minutes and then spun in a cold centrifuge for 10 minutes at 13,000 rpm. The supernatant was collected as the soluble fraction, and the insoluble pellet was twice PBS washed before resuspended and boiled in Laemmli sample buffer. For further fractionation the insoluble pellet was resuspended in CSK buffer with 50 mM NaCl and 1 µl/ml Benzonase, at room temperature for 20 minutes with occasional agitation, before centrifugation. The soluble fraction was retained and the insoluble fraction boiled in Laemmli sample buffer. Whole cell extracts were prepared by lysing cells in NETN buffer (150 mM NaCl, 50 mM Tris (pH 7.5), 5 mM EDTA, 0.5% NP- 40), and incubated on ice before sonication and determining protein concentration.

Generation of PrimPol knockout cell lines in avian DT40 cells

Construction of targeted vectors containing antibiotic resistance cassette was performed via gateway cloning system (Iiizumi et al., 2006) and the primers used summarized in Supplementary Table 2. DT40 cells were electroporated and clones selected as described previously (Sonoda et al., 1998) using histidinol

and puromycin (Sigma) respectively at 1 mg/ml and 0.5 mg/ml. Resistant clones were screened for alleles disruption via Southern blot analysis and RT-PCR using superscript one step kit (Invitrogen), with primers summarized in Supplementary Table 3. Human PrimPol cDNA was sub-cloned into TET inducible vector (adapted from Clontech in Takeda's laboratory, Kyoto University, Japan) containing luciferase gene reporter for clone selection.

DT40 cells were grown at 39°C in RPMI 1640 medium supplemented with 10^{-5} M β- mercaptoethanol, penicillin, streptomycin, 10% foetal calf serum and 1% chicken serum (Sigma), and counted manually on haemocytometer with trypan blue (Invitrogen) staining to assess growth defect. Cell cycle analysis was determined by BrdU labelling and flow cytometry analysis as described previously (Hégarat et al., 2012). Cell viability assay was performed using CellTiter-Blue (Promega) following manufacturer's instructions.

DNA fiber spreading and post replication repair assay

Both techniques were performed as previously described (Edmunds et al., 2008). Post replication repair protocol was adapted to measure DNA replication rate during unperturbed DNA replication and following UV-C irradiation, by pre-labeling cells with ^{14}C-thymidine over two doubling time to normalize the amount of total DNA, and by pulse labeling the cells for 20 minutes with tritium thymidine.

Xenopus egg extract preparation and assays

Demembranated sperm nuclei were prepared by lysolecithin treatment as previously described (Murray et al., 1991). For preparation of interphase Xenopus egg extracts, unfertilized eggs were dejellied, washed and activated with the calcium ionophore A23187 as previously described (Kubota and Takisawa, 1993). Activated eggs were washed in extraction buffer (XB: 10 mM Hepes-KOH, pH 7.7, 100 mM KCl, 0.1 mM $CaCl_2$, 1 mM $MgCl_2$, 50mM sucrose) at 4°C, and then, following removal of excess buffer, crushed by centrifugation at 15,000 x g for 10 minutes. The cytoplasmic layer was supplemented with aprotinin (10 µg/ml), cytochalasin B (50 µg/ml), creatine phosphate (30 mM) and creatine phosphokinase (150 µg/ml) then centrifuged at 60,000 x g for 10 minutes at 4°C (Beckman Optima TLA-55) to generate the replication-competent supernatant fraction. Chromatin isolation was carried out using the method of (Errico et al., 2007) and recombinant GST-geminin prepared as previously described (Stokes and Michael, 2003). PCNA and MCM7 antibodies were both from AbCam while Cdc45 antibody was a generous gift of Vincenzo Costanzo (CRUK Clare hall) and Orc1 antibody, from Dr Julian Blow (Dundee University).

Targeting of the PrimPol locus in mouse ES cells

The *PrimPol* (*CCDC111*) knockout first targeting construct was generated using standard pRed/ET recombineering methods in the IRB Mutant Mouse Core facility. Briefly, the genomic locus of *PrimPol* was captured from a BAC clone (bMQ227f06, Sanger Institute) and screened by PCR. Zeo/pheS and LoxP flanked EM7-Kanamycin cassettes were sequentially recombineered into the BAC up and downstream of exon 5, that contains critical enzymatic residues (AEP I domain), and the modified genomic locus recombineered into a capture vector. The EM7-Kanamycin cassette was deleted by Cre expression, leaving a single LoxP site 3' of exon 5. A Gateway reaction was then carried out to swap the Zeo/pheS cassette for a LacZ/Neo Trap cassette containing a LoxP site upstream of exon 5 (ENSMUSE00001229433) (Supplemental Figure S4C). ES cells were targeted by electroporation of an AsiSI linearized construct followed by selection with Neomycin. Positive clones (*PrimPol*$^{+/trap}$) were screened by long range PCR using DNA isolated from targeted ES cell clones as substrate and Southern blotting was used to confirm single insertion (details available upon request).

Generation of PrimPol deficient mice

Positive *PrimPol*$^{+/trap}$ clones were injected into 3.5 day old mouse blastocysts derived from C57B6/j mice. Approximately 12-15 ES cells were injected into each blastocyst, and injected blasts were re-implanted back into the oviduct of 2.5 day old pseudo-pregnant foster mice. Chimeras born from these injections were scored for chimerism by coat color analysis, and the chimeras showing the highest contribution from the ES cells were mated with C57B6/j wild-type mice. Agouti offspring obtained from these test-matings were screened for the presence of the mutation by PCR. Mice positive for the allele were bred to FlpO (C57BL/6J-Tg(Pgk1-FLPo)10Sykr/J , Jackson Laboratories) transgenic mice to delete the genetrap and generate the floxed allele, *PrimPol*^{cond} (Supplemental Figure S4C). These animals were subsequently bred to Sox2-Cre (Tg(Sox2-cre)#Amc/J , Jackson Laboratories) transgenic mice to delete exon 5 generating the knockout allele, *PrimPol*$^{-}$ (Supplemental Figure S4C). Phenotypic analysis of animals will be described elsewhere. Mice were maintained on a mixed 129/B6 background and all animals were handled in strict accordance with the guidelines of the European Community (86/609/EEC) at the animal facilities in the Barcelona Science Park. The protocols were approved by the Animal Care and Use Committee of the Barcelona Science Park (IACUC; CEEA-PCB) in accordance with applicable legislation (Law 5/1995/GC; Order 214/1997/GC; Law 1201/2005/SG). All efforts were made to minimize suffering.

Generation of mouse embryo fibroblasts lacking PrimPol expression

Mouse embryonic fibroblasts (MEFs) were isolated from *PrimPol*$^{+/-}$ pregnant females at E14.5. Uterine horns were removed and washed with PBS. Embryos were removed and decapitated. Viscera was removed and tail tissue was taken for PCR genotyping (details available upon request). Embryos were washed in fresh PBS and incubated over night at 4ºC in 5 ml trypsin/EDTA. Selected embryos were incubated in trypsin at 37ºC for 20 minutes and physically disaggregated by pipetting in Dulbecco's modified Eagle Medium (DMEM) with 10% fetal bovine serum (FBS), 2 mM L-glutamine and 100 U/ml penicillin/streptomycin (MEF media). Supernatant containing disaggregated cells was plated and washed the following day to remove non-adherent cells. MEFs were cultured in MEF media and were used prior to passage 5 for experiments.

Real time PCR analysis of *PrimPol* mRNA levels

To determine relative mRNA levels of PrimPol, a quantitative Real Time-PCR (qRT-PCR) was developed for the mouse *PrimPol* gene. qRT-PCR was performed using the Step-One-Plus Real Time PCR System (Applied Biosystems) and SYBR Green PCR Master Mix (Applied Biosystems) following manufacturer's instructions. Relative levels were calculated using the comparative CT method. For PrimPol, a forward primer in Exon 5 (taacaaattggccaacccaggag) and a reverse primer in exon 7 (accttagcttcatcatcctcggc) were used. A diagram of the strategy and results run on an agarose gel with a 100 bp size ladder are shown (Supplemental Figure S4D and E). Real time PCR reactions to determine relative levels were performed in triplicate and GAPDH was used as an endogenous control for normalization (Supplemental Figure S4F).

Metaphase chromosome preparations

Mitotic MEF populations were enriched by colcemid (2 x10^{-7} M) treatment for 1 to 2 hours. Cells were harvested by trypsinization, osmotically swollen with 0.075M KCL for 15 minutes at 37ºC and fixed with ice cold methanol:glacial acetic acid (3:1) added dropwise. Cells were resuspended in 300 μl of fixative and 30 μl was spread over a glass slide that was then inverted over an 80ºC water bath for 7 seconds and dried on the hot lid. Slides were stained in 5% Giemsa solution for 10 minutes and examined under oil immersion at 63X-100X magnification, using a white light source. A minimum of 50 spreads was counted per sample and the number of chromosomes per spread and aberrations per spread were recorded. Aphidicolin (Sigma) treatment was performed on primary MEFs cultured in MEF media containing 300 nM aphidicolin for 36 hours.

SUPPLEMENTARY REFERENCES

Engelke, D. R., Krikos, A., Bruck, M. E., and Ginsburg, D. (1990). Purification of *Thermus aquaticus* DNA polymerase expressed in *Escherichia coli*. Anal. Biochem. *191*, 396-400.

Errico, A., Costanzo, V. & Hunt, T. (2007). Tipin is required for stalled replication forks to resume DNA replication after removal of aphidicolin in Xenopus egg extracts. Proc. Natl. Acad. Sci. *104*, 14929-14934.

Evans, S.J., Fogg, M.J., Mamone, A., Davis, M., Pearl, L. H. & Connolly, B.A. (2000) Improving dideoxynucleotide-triphosphate utilisation by the hyper-thermophilic DNA polymerase from *Pyrococcus furiosus*. Nucleic Acids Res. *28*, 1059–1066.

Hégarat, N., Smith, E., Nayak, G., Takeda, S., Eyers, P. A., & Hochegger, H. (2012). Aurora A and Aurora B jointly coordinate chromosome segregation and anaphase microtubule dynamics. J. Cell Biol. *195*, 1103-13.

Iiizumi, S., Nomura, Y., So, S., Uegaki, K., Aoki, K., Shibahara, K., Adachi, N., & Koyama, H. (2006) Simple one-week method to construct gene targeting vectors: application to production of human knockout cell lines. BioTechniques *41*, 311-316.

Jozwiakowski, S. K., & Connolly, B. A. (2011). A modified family-B archaeal DNA polymerase with reverse transcriptase activity. Chembiochem *12*, 35-7.

Stokes, M. P., & Michael, W. M. (2003). DNA damage-induced replication arrest in Xenopus egg extracts. J Cell Biol. *163*, 245-55.

Zlatanou, A., Despras, E., Braz-Petta, T., Boubakour-Azzouz, I., Pouvelle, C., Stewart, G. S., Nakajima, S., Yasui, A., Ishchenko, A. A., Kannouche, P. L. (2011). The hMsh2-hMsh6 Complex Acts in Concert with Monoubiquitinated PCNA and Pol eta in Response to Oxidative DNA Damage in Human Cells. Mol. Cell *43*, 649–662.
